# Perceived organizational support and moral distress among nurses

**DOI:** 10.1186/s12912-017-0270-y

**Published:** 2018-01-10

**Authors:** Navideh Robaee, Foroozan Atashzadeh-Shoorideh, Tahereh Ashktorab, Ahmadreza Baghestani, Maasoumeh Barkhordari-Sharifabad

**Affiliations:** 1 Student Research Committee of Nursing and Midwifery, International Branch of Shahid Beheshti University of Medical Sciences, Tehran, Iran; 2 Department of Nursing Management, School of Nursing and Midwifery, Shahid Beheshti University of Medical Sciences, Vali-Asr Avenue, Cross of Vali-Asr and Hashemi Rafsanjani Highway, Opposite to Rajaee Heart Hospital, Tehran, 1996835119 Iran; 3 School of Nursing and Midwifery, Shahid Beheshti University of Medical Sciences, Tehran, Iran; 4 Department of Biostatistics, School of Allied Medical Sciences, Shahid Beheshti University of Medical Sciences, Tehran, Iran; 5 Department of Nursing, School of Medical Science, Yazd Branch, Islamic Azad University, Yazd, Iran

**Keywords:** Ethics, Morals, Perceived organizational support, Moral distress, Nurses

## Abstract

**Background:**

Moral distress is prevalent in the health care environment at different levels. Nurses in all roles and positions are exposed to ethically challenging conditions. Development of supportive climates in organizations may drive nurses towards coping moral distress and other related factors. This study aimed at determining the level of perceived organizational support and moral distress among nurses and investigating the relationship between the two variables.

**Methods:**

This was a correlational-descriptive study. A total of 120 nurses were selected using random quota sampling method. A demographic questionnaire, Survey of Perceived Organizational Support, and Moral Distress Scale were used to collect the data which were analyzed using descriptive and analytical tests in SPSS20.

**Results:**

The mean perceived organizational support was low (2.63 ± 0.79). The mean moral distress was 2.19 ± 0.58, which shows a high level of moral distress. Moreover, Statistical analysis showed no significant relationship between perceived organizational support and moral distress (*r* = 0.01, *p* = 0.86).

**Conclusion:**

Given the low level of perceived organizational support and high moral distress among nurses in this study, it is necessary to provide a supportive environment in hospitals and to consider strategies for diminishing moral distress.

## Background

Moral distress is a common problem among the professionals employed in health care settings [1]*.* It occurs when the individuals feel that they cannot act according to the pivotal values and duties or when the measures taken to achieve the intended results fail to succeed so that the totality of individual’s ethical principles is seriously endangered [2]. In other words, moral distress can be considered as the stress tolerated by professionals at a time when, despite an awareness of the right performance, they cannot achieve the correct performance due to some barriers [1, 3–5]. Moral distress has been described as a major problem in the nursing profession [5, 6].

The particular characteristics of nursing and the different work cultures generated in different health care institutions expose nurses to a higher risk of moral distress than other professionals [7]. Moral distress has undesirable outcomes for both nurses and patients, and can have direct and indirect effects on nurses. Physical disorders such as nightmares, headache and anxiety and a dysfunctional personal life have been reported among nurses at risk for moral distress [8].

Moreover, feeling of anger, failure, sin, and disability are among the consequences of moral distress. Some studies have demonstrated that moral distress is correlated with personnel burnout, deteriorated team work, reduced quality of care, and challenges related to patient safety [2, 9, 10]. It will further lead to occupational stress and turnover [11].

Various factors contribute to moral distress in nurses among them are invasive procedures on patients with incurable diseases, orders for unnecessary tests or examinations, insufficient and inefficient treatment by colleagues, lack of balance in power among the health specialists, and lack of organizational support [12]*.*

When exposed to stressful environments and high job demands, the employees need the financial and spiritual support of their organization. Perceived organizational support is a condition based on how much the organization considers employees’ values and needs [13]. Organizational support is one of the important indices of nursing work environment [14], and can be considered an influential moral factor [15].

Additionally, one aspect of ethical competency of nurse leaders is their supportive behavior [14]. The leaders’ supportive behavior will be compensated for by the followers’ proper compliance. The organization is less likely to face a situation in which the staff’s behavior disturbs the leader’s or group’s work [14, 16–20]. This perception of level of organizational support especially about ethical practice is a vital element of the constraints upon nurses’ actions [21].

Perceived organizational support reduces stressors in the workplace and is potentially involved in dealing with work-related fatigue, excitement, and depression [22]. Supportive occupational environments are the most important factor in creating job satisfaction for nurses that influences positively the patients’ treatment, absorption and maintenance of manpower in the organization. A climate with high levels of support diminishes occupational tension and maintains the nurses in the organization [23].

Moreover, some studies have revealed that perceived organizational support is negatively correlated with work absenteeism [24], and intent to turnover [25], while it is positively correlated with award expectation, role of performance and social behavior, preventive and civil behaviors [26], and also organizational commitment and subsequently self-competency [27]. Nurse leaders’ supportive behavior plays a key role in productivity and promoting nurses’ professional performance [20, 28].

Iran is a developing country located in the south-west of Asia with a population of about 80,000,000 people. The nursing manpower at different levels is estimated to be about 150,000. As it is the case in many other developing countries, nurses in Iran encounter many challenges such as long working hours, changing work shifts, limited vacation, abundant occupational wants and wishes, unsatisfactory payment or salary, and inappropriate behavior towards some patients or their families [29, 30]. These challenges often result from deficient techniques of manpower management in hospitals [30], shortage of manpower, job dissatisfaction, nurses’ low social status, absence of a satisfactory student acceptance system at the universities, and shortage of ethics course in the nursing curriculum [31–36], leading to increased workload, fostered medical and nursing errors, and subsequently, moral distress in nurses [36].

There are some controversies about the level of moral distress among Iranian nurses [37–40]. A review of literature related to the two variables “perceived organizational support” and “moral distress” indicated that the investigation of these two variables has been very limited on Iranian nurses. Furthermore, no comprehensive study has been found on determining the correlation between “perceived organizational support” and “moral distress” among Iranian nurses. Development of supportive organizations may lead nurses to better cope with moral distress and other problems such as job dissatisfaction [5, 21, 41–43].

### Aim

The purpose of this research was to determine the level of perceived organizational support and moral distress among nurses and to investigate the relationship between these two variables.

## Methods

### Research design

This correlational descriptive study used random quota sampling to select the participants. First, considering the distribution of hospitals affiliated to Shahid Beheshti University of Medical Sciences in different regions of Tehran (north, south, east, west and center), one hospital was randomly selected from each region and the required number of nurses was selected from each hospital in proportion to the total number of nurses working in it. Considering the number of hospitals surveyed, 120 questionnaires were distributed among all the qualified nurses selected from the morning, evening, and night shifts using random sampling.

The sample size was calculated by the following formula to explore correlation between moral distress and perceived organizational support.

$$ N={\left[\frac{Z_{\alpha }+{Z}_{\beta }}{c}\right]}^2+3 $$

Where N is the desired sample size, *Z*_*α*_ is the standard normal score of 95% of confidence interval = 1.96, *Z*_*β*_ = statistical power at 90%, which is 1.28 and *c* = 0/5 × *Ln*[(1 + *r*)(1 − *r*)] with being the correlation coefficient, which is 0.3 according to a study by Jay Maningo-Salinas [15].

Considering a participant attrition of 10%, 120 nurses were selected for the study. However, 110 completed questionnaires were analyzed. The study inclusion criteria consisted of having a bachelor’s degree or higher in nursing and at least 1 year of work experience.

### Data collection tools

In this study, a demographic questionnaire, Eiesenberger’s Survey of Perceived Organizational Support (SPOS), and nurses’ Moral Distress Scale (MDS) were used to collect the data.

#### Demographic information questionnaire

The demographic questionnaire examined participants’ demographic data including age, gender, marital status, level of education, work experience, work shifts, and history of attendance in ethics workshops.

#### *Survey* of *Perceived Organizational Support*

The 8-item SPOS was developed by Eisenberger et al. [13]. Each item in this survey is scored based on a 7-point Likert scale from strongly disagree (zero) to strongly agree (six). The range of scores in each item varies from zero to six while on a total scale is zero to 48. A higher score indicates more perceived organizational support. This scale is a unidimensional measure and has been widely used in research studies. Evidence of its validity and reliability has been reported in numerous studies [44–46]. The Cronbach’s alpha coefficient of perceived organizational support was calculated as 0.74 in this study.

#### Moral distress scale

The nurses’ MDS is a native scale developed by Atashzadeh-Shoorideh et al. [40]. It contains 30 items in three dimensions, namely “inappropriate competencies and responsibilities”, “errors”, and “not respecting the ethical principles”. All the items in this scale are scored based on a 5-point Likert scale from 0 (not at all) to 4 (very much). The score of moral distress is then calculated as the mean of the total score of the items. The score of moral distress obtained is then classified into four categories: 0–1 is low, 1.01–2 is moderate, 2.01–3 is high, and 3.01–4 is very high moral distress. The Cronbach’s alpha coefficient for the “Moral Distress Scale” and all of its dimensions designed by Atashzadeh-Shoorideh et al. was calculated in this study as 0.77.

### Data collection

The participants were oriented on how to answer the questionnaires and were informed about the voluntary nature of participation in the study. The questionnaires were distributed among nurses working in different shifts and were collected within 2 days.

### Data analysis

The collected data was analyzed via SPSS 20 using the descriptive statistics of data as absolute and relative frequency report, and inferential statistics as a determination of correlation between the variables under study via Pearson product moment correlation coefficient.

## Results

The study participants consisted of 110 nurses with a mean age of 34.1 ± 7.4 years and a mean work experience of 9.6 ± 6.5 years, 90% of them were female, 55.5% were married and 95.5% held a bachelor’s degree in nursing. The majority of the nurses (48.2%) were working in rotating shifts. The majority (51.8%) had not attended ethics workshops in the past (Table 1).

**Table 1:** Sociodemographic characteristics of study participant

Variables		M (SD)	n (%)
Age		34.1 (7.4)	
Gender	Female		99 (90)
	Male		11 (10)
Marital Status	Single		49 (44.5)
	Married		61 (55.5)
Level of Education	Bachelor’s Degree		105 (95.5)
	Master’s Degree		5 (4.5)
Work Experience		9.6 (6.5)	
Work Shifts	Fixed		35 (31.8)
	Rotating		75 (68.2)
History of attendance in ethics workshops.	Yes		53 (48.2)
	No		57 (51.8)

As shown in Table 2, the mean perceived organizational support was low (2.63 ± 0.79) and the mean moral distress was high (2.19 ± 0.58). The highest mean of moral distress pertained to the dimension of errors (2.43 ± 0.65).No relationships were observed between perceived organizational support and moral distress (*p* = 0.86) or its dimensions (*p* > 0.05); (Table 3).

**Table 2:** Mean and standard deviation of perceived organizational support, moral distress, and the associated dimensions

Variable	Mean(SD)	Range of Score
Perceived organizational support	2.63 (0.79)	0.75–4.25
Total moral distress	2.19 (0.58)	1–3.40
Inappropriate competencies and responsibilities	2.12 (0.54)	1–3.70
Errors	2.43 (0.65)	1–3.73
Not respecting the ethical principles	1.96 (0.76)	1–3.56

No relationships were observed between perceived organizational support and moral distress (*p* = 0.86) or its dimensions (*p* > 0.05); (Table 3)

**Table 3:** Correlation between perceived organizational support and moral distress and its dimensions

	Inappropriate competencies and responsibilities		Errors		No respect for ethics		Total moral distress	
	r	p	r	p	r	p	r	p
Perceived organizational support	0.048	0.61	0.062	0.52	−0.062	0.52	0.017	0.86

There was a statistically significant relationship between moral distress and work shifts. Also, relationship between the dimension of errors and work shifts was statistically significant. The significance level set for the work shift test was *p* = 0.04 for moral distress and *p* = 0.00 for the dimension of errors. A significant relationship was also observed between the inappropriate competencies and responsibilities dimension of moral distress and work experience (*p* = 0.04) as shown in Table 4.

**Table 4:** Correlation between organizational support and moral distress and its dimensions with Sociodemographic characteristics

Variable		Perceived organizational support		Total moral distress		Inappropriate competencies and responsibilities		Errors		Not respecting the ethical principles	
		r	p	r	p	r	p	r	p	r	p
Age		−0.10	0.25	−0.04	0.67	0.12	0.20	−0.17	0.07	0.00	1.00
Gender	Female	0.01	0.89	0.06	0.51	0.07	0.43	0.06	0.54	−0.03	0.70
	Male										
Marital Status	Single	0.07	0.42	−0.07	0.42	0.08	0.40	−0.17	0.06	−0.01	0.86
	Married										
Level of Education	Bachelor’s Degree	0.10	0.27	0.10	0.28	0.18	0.06	0.08	0.40	0.76	0.43
	Master’s Degree										
Work Experience		−0.14	0.12	0.00	0.98	0.19	0.04^*^	−0.14	0.13	0.00	0.95
Work Shifts	Fixed	0.07	0.44	0.18	0.04^*^	0.07	0.41	0.25	0.00^*^	0.13	0.15
	Rotating										

^*^p < 0.05

## Discussion

This study was conducted to determine the level of perceived organizational support and moral distress among nurses and to investigate the relationship between these two variables.

The results revealed low perceived organizational support in the nurses, this finding supports the results of previous studies. In a study by Kwak conducted on nurses in South Korea, organizational support was investigated using corrected nursing work index with a rate assessed as falling in the low limits [47]. Another study carried out on nurses in Italy reported the mean score of perceived organizational support as 2.26 ± 0.78 which is lower than the central point value reported by Eisenberger et al.’s scale [48]. This is inconsistent with other studies that reported moderate perceived organizational support [15, 29]. Jay Maningo-Salinas investigated the perceived organizational support level among oncology nurses using Eisenberg et al.’s scale and reported it at the moderate level of 3.70 ± 0.86.16. Moreover, the study by Gorji et al. reported the perceived organizational support among the emergency room nurses as moderate [29].

The dissimilarity of results between these studies and the present one may be due to the differences in the instruments used, the research populations, the climate of the organization and how their respective managers managed the research populations. The lack of a supportive environment in hospitals may cause further moral and work conflicts, job dissatisfaction, and reduced employees’ trust in the organization [48].

The current study reported the intensity of moral distress as high among the nurses, which is consistent with the results obtained by Woods et al. conducted on nurses in New Zealand [49]. Also, Cummings found that the prevalence of moral distress was high among nurses and proposed this phenomenon as responsible for nurses’ turnover rates [50].

Moral distress has been reported as moderate in some studies [37–39, 53], while it is reported to be lower than moderate in a number of other studies [51, 52]. A study on Swedish nurses elucidated the point that moral distress was at the low range [54]. Additionally, the studies carried out in America reported the nurses’ moral distress scores at rather low levels [5, 55]. Another study undertaken in Turkey showed that nurses had low-level moral distress [1], a finding which is inconsistent with our results.

This inconsistency may be attributed to differences in organizational, cultural, educational, geographical, and individual factors and beliefs. For instance, mention can be made of the existence of the required standards of care in hospitals, level of knowledge and awareness, high participation of the health staff, and ethical traits of the participants. Also, it may be speculated that the extreme differences in moral distress between this study and other endeavors may be attributed to variations in the study population and measurement instruments used in the present study. The scale used in this study to measure moral distress includes three dimensions while the moral distress instrument used in other studies has been one-dimensional with some items not appropriately working in the Iranian context. For example, the item of “discharge a patient when he has reached the maximum length of stay based on diagnostic related grouping although he has many teaching needs” in Corley Moral Distress Scale isn’t appropriate in Iranian nurses. Comparing the findings of this research with other studies has shown that moral distress for most nurses is moderate to high.

In this study, the highest level of moral distress pertained to the dimension of “errors”, while the lowest level belonged to the aspect of “not respecting ethical principles”. In numerous studies, the most common causes of moral distress among nurses have been reported to be working with incompetent staff [1, 40, 49, 56], useless care [1, 40], and inappropriate intra-team relations [1, 49]. On the basis of the results of these studies, high level of distress in the dimension of “errors” seems to be logical compared to other dimensions.

The results of this study showed no statistically significant relationships between perceived organizational support and moral distress and any of its’ dimensions, A study by Maningo-Salinas, conducted on oncology nurses, expunged upon the correlation between moral distress and inclination for turnover and also determined the mediating effect of perceived organizational support on these two variables. Their findings suggested that perceived organizational support does not mediate the correlation between moral distress and inclination for turnover and that the interaction between moral distress and perceived organizational support was not statistically significant [15]. They point out no statistically significant relationship, this possibility suggests that Eisenberg’s perceived organizational support scale may not be the best instrument for measuring perceived organizational support among the nurses. Hence, it is necessary to carry out a more comprehensive study regarding the use of a perceived organizational support tool in nursing [15].

Of course, several studies have reported the effect of ethical work climate on moral distress. The ethical climate does not often lead to personnel’s’ perceived organizational support. The reason for this can be the creation of a work environment which is reliable in the organization [57]. Fogel showed that ethical climate agents have a moderating effect on the moral distress, turnover, poor patient care, and justice subjects [58]. In the study by Fogel, the relations between managers and Fnurses induced significant effects on ethical work climate [58]. This, in turn, affects moral distress. Silen’s study showed that ethical climate is an important factor in nurses’ work setting [54]. But in some articles, it has been indicated that a negative relationship exists between ethical climate and moral distress [50–53, 59].

In the current study, perceived organizational support was not significantly correlated with any of the demographic variables examined, while a significant relationship was observed between the inappropriate competencies and responsibilities dimension of moral distress and the variable of work experience. The present study found a statistically significant relationship between total moral distress and the dimension of errors and work shifts. This is inconsistent with the results obtained by Atashzadeh-Shoorideh et al. [40]. The inconsistency between the findings of the present study and the study of Atashzadeh-Shoorideh et al. may be due to the differences in the research setting and work environment, which may have led to lower rates of error and moral distress in the nurses examined by Atashzadeh-Shoorideh et al. [40].

The limitations of this research are the descriptive design and data collection with a questionnaire and reliance on self-report data. As a result, some people may refuse to provide real responses and give unrealistic responses. A further limitation is the potential impact of confounding factors such as high occupancy, fatigue, and lack of readiness of nurses to complete the questionnaire. This study used a cross-sectional design. For this reason, it makes the conclusion about cause-effect relations difficult. Therefore, a closer examination can be done by conducting in-depth and longitudinal studies. A further limitation of this research was the selection of nurses from just one city. With the implementation of national and international studies, the possibility of generalizing these findings will increase.

## Conclusion

The results of this study showed that the level of perceived organizational support was low in nurses and moral distress was high. Therefore, it is necessary to provide a supportive environment in hospitals and to consider strategies for diminishing moral distress.

Also, the findings indicated that there was no significant correlation between perceived organizational support and moral distress. These results are not consistent with the findings of other studies on moral distress. It is recommended that a similar study be carried out with other measurement scales of organizational support and the results be compared and contrasted with our findings.

## Abbreviations

ICU: Intensive Care Unit
MDS: Moral Distress Scale
RN: Registered Nurse
SPOS: Survey of Perceived Organizational Support
